# 100 million years of multigene family evolution: origin and evolution of the avian MHC class IIB

**DOI:** 10.1186/s12864-017-3839-7

**Published:** 2017-06-13

**Authors:** Julien Goebel, Marta Promerová, Francesco Bonadonna, Karen D. McCoy, Céline Serbielle, Maria Strandh, Glenn Yannic, Reto Burri, Luca Fumagalli

**Affiliations:** 1 Laboratory for Conservation Biology, Department of Ecology and Evolution, University of Lausanne, Biophore Building, CH-1015 Lausanne, Switzerland; 2 Institute of Vertebrate Biology of the Czech Academy of Sciences, Kvetna 8, 60365 Brno, Czech Republic; 3 Present address: Max Planck Institute for the Science of Human History, Kahlaische Strasse 10, D-07745 Jena, Germany; 4 CNRS, UMR 5175, Centre for Functional and Evolutionary Ecology, F-34293 Montpellier, France; 5 MIVEGEC UMR 5290 CNRS-IRD University of Montpellier, Centre IRD, F-34394 Montpellier, France; 6 Present address: Molecular Ecology and Evolution Lab, Department of Biology, Lund University, Sölvegatan 37, SE-223 62 Lund, Sweden; 7 LECA – Laboratoire d’Écologie Alpine, UMR CNRS 5553, Université Savoie Mont Blanc, F-73376 Le Bourget-du-Lac, France; 8 Department of Population Ecology, Institute of Ecology, Friedrich Schiller University Jena, Dornburger Strasse 159, D-07743 Jena, Germany

**Keywords:** Birds, Birth-death evolution, Concerted evolution, Gene duplication, Gene conversion, Major histocompatibility complex, Recombination

## Abstract

**Background:**

Gene duplication has led to a most remarkable adaptation involved in vertebrates’ host-pathogen arms-race, the major histocompatibility complex (MHC). However, MHC duplication history is as yet poorly understood in non-mammalian vertebrates, including birds.

**Results:**

Here, we provide evidence for the evolution of two ancient avian MHC class IIB (MHCIIB) lineages by a duplication event prior to the radiation of all extant birds >100 million years ago, and document the role of concerted evolution in eroding the footprints of the avian MHCIIB duplication history.

**Conclusions:**

Our results suggest that eroded footprints of gene duplication histories may mimic birth-death evolution and that in the avian MHC the presence of the two lineages may have been masked by elevated rates of concerted evolution in several taxa. Through the presence of a range of intermediate evolutionary stages along the homogenizing process of concerted evolution, the avian MHCIIB provides a remarkable illustration of the erosion of multigene family duplication history.

**Electronic supplementary material:**

The online version of this article (doi:10.1186/s12864-017-3839-7) contains supplementary material, which is available to authorized users.

## Background

Gene duplication represents an important source of evolutionary novelties and has led to outstanding adaptations, such as the vertebrates’ adaptive immune system (e.g. [1–3]). Genes of the major histocompatibility complex (MHC) take a prominent role in the latter, as they are strongly associated with individual fitness, and have been instrumental for understanding the evolution of multigene families. The duplication history and mode of evolution of the MHC have been debated over decades [2, 4–8], and remain obscure for major vertebrate classes, such as birds. To clarify the phylogenetic origins and evolutionary history of this important component of the avian immune system, analyses of MHC diversity across the entire avian tree of life have been called for [9].

The MHC multigene family was originally thought to evolve under concerted evolution [10], whereby gene conversion exchanges sequence information among paralogs (i.e. duplicate genes) and thereby homogenizes the sequence content across the multigene family. However, phylogenetic reconstructions showed that mammalian MHC sequences cluster by locus (i.e. according to duplication history) rather than by species (e.g. [7]). Together with the phylogenetically scattered loss of MHC lineages (e.g. [11, 12]) this observation suggested that the mammalian MHC rather follows a birth-death process, in which the dynamics of gene duplication (birth) and gene loss (death) are important determinants of the multigene family’s long-term evolution [10].

In contrast, phylogenetic evidence for birth-death evolution has emerged only recently from the avian MHC class IIB (MHCIIB) [9, 13]. Initial phylogenetic reconstructions of MHC diversity in fowl (Galliformes) and songbirds (Passeriformes) found mostly species-specific sequence clusters, leading to the conclusion that the avian MHC evolves under concerted evolution [14–18]. Later studies in these orders (e.g. [19, 20]) and in birds of prey (Accipitriformes) [13, 21] confirmed these patterns; though predominantly for exon 2 (see Additional file 1 for gene structure), which is involved in the binding of pathogen-derived peptides and evolves under strong balancing selection [22]. However, the finding of two orthologous sequence clusters (*DAB1* and *DAB2*) in owls (Strigiformes) started casting a different light. Based on a sequence signature comprised of 16 divergent sites scattered across the 5′-end of exon 3, duplication history was traced beyond the owl order to charadriiform birds [13], and subsequently to the root of the Neoaves radiation [9], confirming the persistence of two avian MHCIIB lineages over at least 70 million years (my) [23]. Also in other bird orders, including tubenoses (Procellariiformes) and even passerines (Passeriformes), indications for divergently evolving MHC paralogs are accumulating [24–27]. Together with the supposed repeated loss of MHC lineages [9] and mammal-like MHC organizations in some bird species [24, 25], these results suggest that birth-death processes may constitute an important component of not only mammalian but also avian MHC evolution.

Still, the time of origin of the two avian MHCIIB lineages and the potential role of concerted evolution in concealing it remains unknown. To perform a systematic survey of MHCIIB lineages across the avian tree of life, we isolated avian MHCIIB sequences spanning from exon 1 to exon 4 with an unprecedented phylogenetic coverage [28]. Based on phylogenetic analyses of these data along with sequences available from DNA sequence databases, we (i) determined the phylogenetic origin of the two avian MHCIIB lineages, and (ii) studied their evolution across the avian tree of life.

## Results and discussion

### The origin of avian MHCIIB lineages predates the radiation of extant birds

**Fig. 1:**
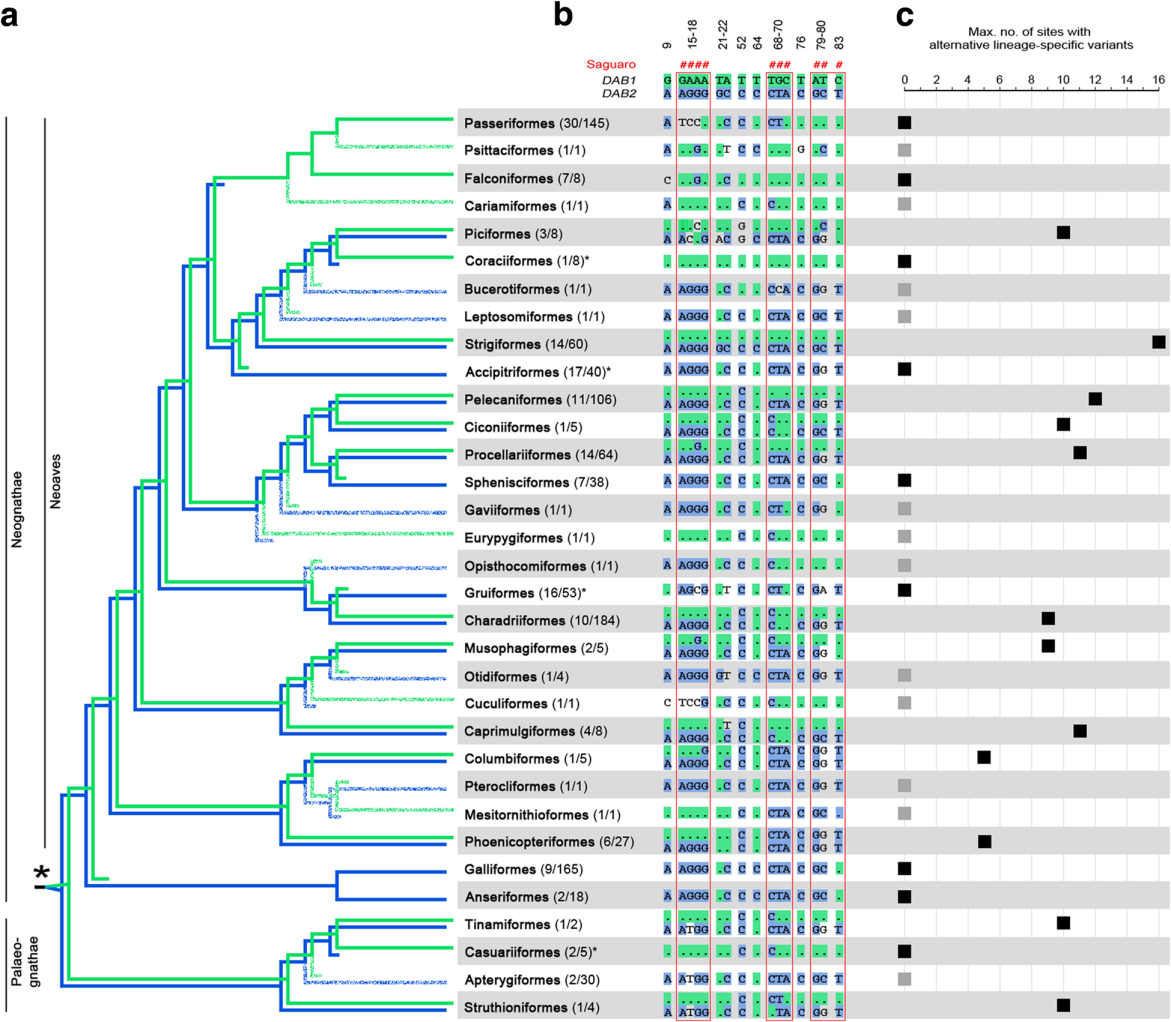
**a** Schematic of the phylogenetic distribution of the avian *DAB1* (*green*) and *DAB2* (*blue*) MHCIIB lineages based on the phylogenetic placing of sequences (Fig. 2, Additional file 1) and on the screening of sequence signatures for *DAB1*- and *DAB2*-specific variants (panel **b**). The duplication event preceding the avian radiation is depicted by an *asterisk*. In parentheses, the number of species and sequences are provided for each order. *Asterisks* following *parentheses* indicate orders for which only one lineage was identified despite efforts to isolate the second lineage [28]. Accordingly, for orders with only a single lineage available on DNA databases obtained with non-significant sequencing efforts, branches are presented in dissolved colors. **b** Alignment of the 16 lineage-specific sites in exon 3. For each order, the most *DAB1*-like and the most *DAB2*-like sequence is presented. *Dots* in the alignment represent variants identical with the *DAB1* sequence signature. *Green* and *blue* shading indicate variants identic with the *DAB1*-specific and *DAB2*-specific variants, respectively. Sites identified to trace duplication history using Saguaro are highlighted with hashes and red frames. **c** Degrees of divergence/homogenization of the lineages within avian orders. For each order, the number of pairwise differences between the most *DAB1*-like and the most *DAB2*-like sequences within the 16-bp signatures is provided. For orders with only one type of sequence, this number is zero and is indicated in *grey* where only a single sequence was available (usually from a genome assembly)

**Fig. 2:**
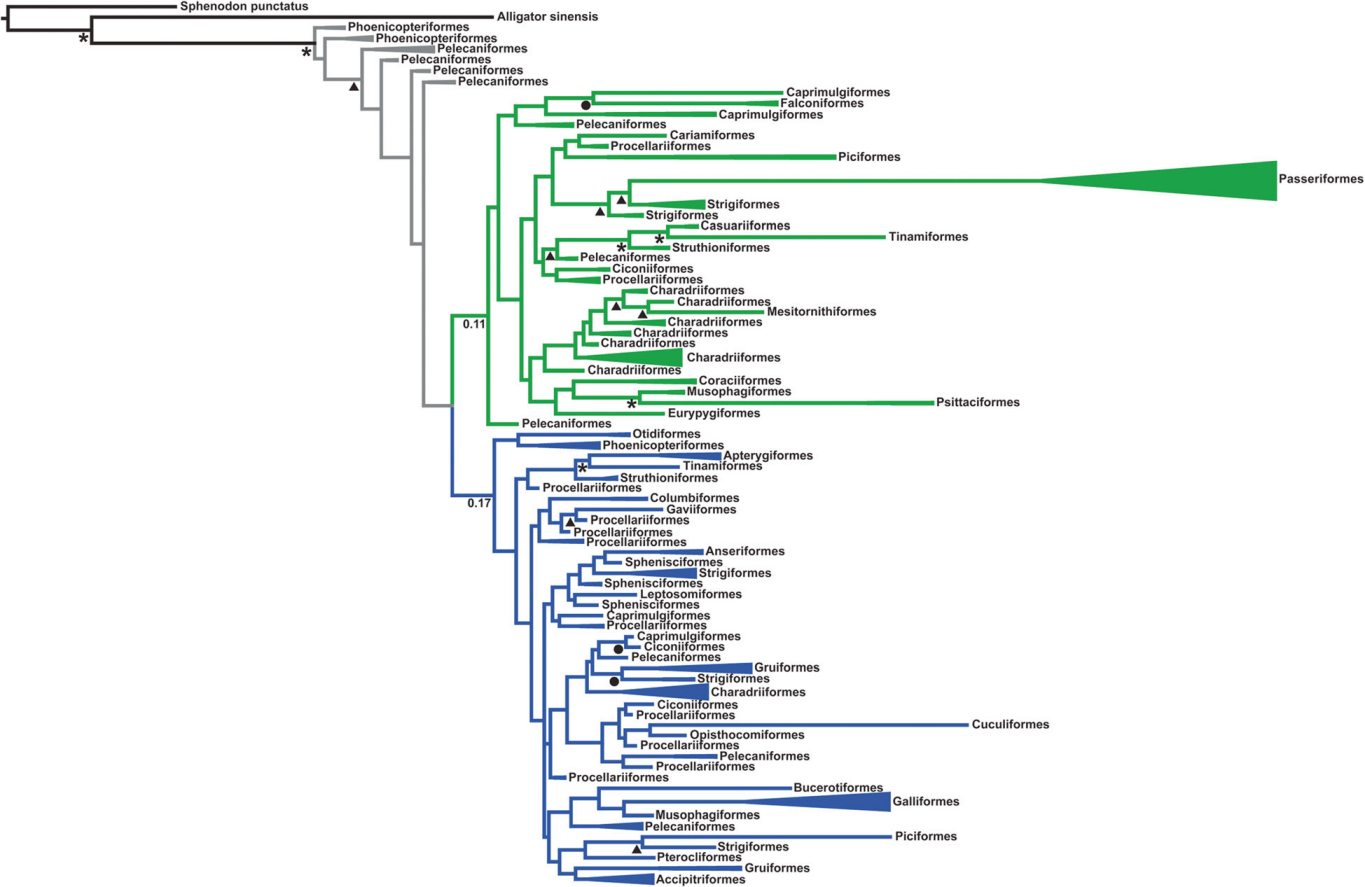
Phylogenetic relationships of MHCIIB exon 3 sequences (first 220 bp). The two monophyletic clusters comprising sequences with predominantly *DAB1* signature or *DAB2* signature are shown in *green* and *blue*, respectively. *Grey* colored branches lead to sequences from pelecaniform and phoenicopteriform species with strongly intermingled lineage signatures. Sequence clusters with sequences from the same order have been collapsed for reasons of readability. A fully resolved topology is provided in Additional file 1. Symbols represent Bayesian posterior probabilities of the resolved-consensus tree: *asterisks*, ≥0.95; *circles*, 0.8–0.9; *triangles*, 0.5–0.8

We found that the two avian MHCIIB lineages evolved prior to the radiation of all extant birds. The screening of sequence data of 175 species from 33 orders for the presence of the sequence signatures in MHCIIB exon 3 [9, 13] revealed the presence of variants characteristic of both MHCIIB lineages in twelve orders across the entire avian phylogeny (Fig. 1a, b). In support of this result, phylogenetic analyses placed the exon 3 sequences of species from ten of these orders into two separate clusters (Fig. 2, Additional file 2) corresponding to the previously described MHCIIB lineages (note that previous analyses excluded functional convergence as a cause for the clustering by locus; [9]). The grouping of sequences from the same order (and species) in two different clusters was confirmed by a phylogenetic network (Additional file 3), even though the network displayed highly reticulate relationships with the major split separating passerine sequences from all other sequences (in line with the long branch leading to this order identified in a previous study [9]). The same relationships were recovered by phylogenetic analyses restricted to the 16 sites previously identified to trace duplication history (Additional file 4), with the two MHCIIB lineages clearly separated also in phylogenetic networks (Additional file 5). Most importantly, all analyses confirmed the presence of both MHCIIB lineages for both neognaths and palaeognaths. (Figs. 1 and 2, Additional files 2, 3, 4 and 5), unambiguously dating the duplication event leading to the evolution of the two avian MHCIIB lineages prior to the radiation of extant birds >100 mya [23].

We next investigated whether sites other than the 16 originally described ones may reflect avian MHCIIB duplication history, and found that this is not the case. To identify sets of sites with a common phylogenetic history, we implemented a hypothesis-free algorithm that reconstructs site-wise phylogenetic relationships (Saguaro; [29]). Saguaro recovered two major types of topologies when run along an alignment including exon 2 and exon 3. The first split sequences from a given species up into two separate clusters (Additional file 6A) with a high distance between the most distant sequences of this species (Additional file 7), as expected for sites that recover duplication history. The second rather grouped sequences by species/order (Additional file 6B), with short distances between the most distant sequences of a given species (Additional file 7), as expected under concerted evolution. This approach identified ten sites that discriminate between the two sequence clusters and thus reflect duplication history (Fig. 1b, Additional files 8 and 9). These sites are a subset of the original 16 sites and recovered the duplication history reflected by entire exon 3 (Additional files 8 and 9). Variants at the six sites not recovered by Saguaro are present also in several orders across the phylogeny (Fig. 1b). Likely, the footprints of duplication at these sites were overwhelmed by the reticulate phylogenetic signals generated by concerted evolution (see below).

### Concerted evolution erodes the footprints of avian MHCIIB duplication history and may mimic birth-death evolution

The phylogenetic distribution of the MHCIIB lineages may hint towards multiple independent losses of both MHCIIB lineages during the radiation of extant birds. According to phylogenetic reconstructions, a significant proportion of orders exhibit only one of the MHCIIB lineages (nine when only orders with significant sequencing efforts are included; 21 when including all orders). This scattered pattern of presence and absence of lineages is a hallmark of birth-death evolution [10] also observed in mammals [7, 30]. Assuming that the isolation of MHCIIB sequences did not miss lineages in many orders (an invalid assumption e.g. for orders with only a single MHCIIB exon 3 sequence available from genome assemblies; Fig. 1), this result might suggest that each lineage was lost multiple times independently.

**Fig. 3:**
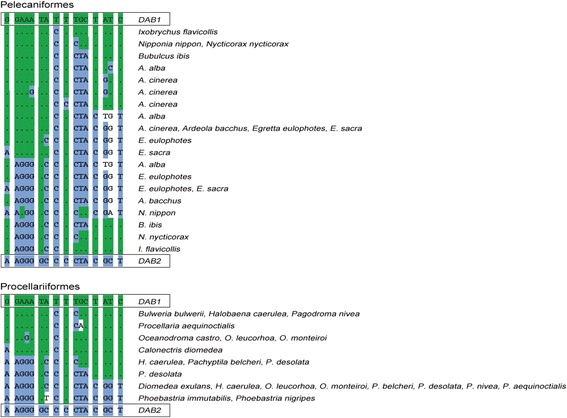
Intermingling of MHCIIB lineage-specific signatures by concerted evolution in pelecaniform and procellariiform birds. *Green shading* and *dots* in the alignment indicate identity with the *DAB1* signature. *Blue shading* indicates identity with the *DAB2* signature

However, our results suggest that more likely in many orders the presence of both MHCIIB lineages has been masked by concerted evolution. Under concerted evolution, intergenic gene conversion transfers sequence information among gene family members [31], and can thereby intermingle and homogenize sequence signatures characteristic of different lineages. The screening for the two MHCIIB lineages revealed two striking findings that illustrate such an impact of concerted evolution on the long-term evolution of the avian MHCIIB region. First, in many species, signatures were intermingled relative to the ones in owls (or vice versa) (Figs. 1b and 3) – concerted evolution appears to have reshuffled the variants distinguishing the two MHCIIB lineages into new exon 3 haplotypes. This intermingling is also reflected in the highly reticulate structure of phylogenetic networks (Additional files 3, 5, and 9). The intermingling of single variants within relatively short sequence stretches suggests that concerted evolution occurred through gene conversion events involving short sequence tracts. In some orders, such as Pelecaniformes and Phoenicopteriformes, this process appears to have resulted in an entire collection of haplotypes, with multiple haplotypes displaying various degrees of intermingling between *DAB1* and *DAB2* signatures (Fig. 3). Second, the number of lineage-specific sites retained varies considerably among species and orders (Fig. 1c). From a functional perspective, these results suggest that variants at the originally divergent sites are largely interchangeable, implying that a functional divergence of the two lineages is unlikely. From a phylogenetic perspective, the reticulate sequence evolution and erosion of lineage signatures implied by these results is expected to hinder the reconstruction of the duplication history, as reflected by several of our results: statistical supports for phylogenetic relationships are low; and recombinant sequences are placed in the cluster for which they exhibit more lineage-specific variants (e.g. in Columbiformes) or at the base of the two lineages when proportions of lineage-specific variants are about equal (grey branches leading to Pelecaniformes and Phoenicopteriformes) (Fig. 2, Additional file 2). These results illustrate how the homogenization and loss of sequence signatures may ultimately erase duplication history. In the avian MHCIIB, the presence of a range of intermediate evolutionary stages along this process, therefore, provides a remarkable demonstration of how the erosion of the footprints of gene duplication history by concerted evolution advances on the long term.

Finally, our results suggest that, in many orders for which phylogenetic relationships would postulate the presence of only a single MHCIIB lineage, the two lineages might indeed be present despite the presence of only one lineage-specific signature. Careful inspection of the composition of avian MHCIIB lineages’ signatures reveals many sequences with signatures composed of variants from both lineages (Fig. 1b). Even orders such as passerines and galliforms, in which phylogenetic analyses identified only a single lineage despite a well-characterized MHCIIB (e.g. [32, 33]), exhibit single sites within the 16-bp signature with variants characteristic of the alternative lineage (Fig. 1b). Consequently, instead of multiple independent losses of avian MHCIIB lineages, in many avian orders the presence of the two MHCIIB lineages may have been masked by concerted evolution.

The retention time of signatures of each MHCIIB lineage across bird species is thus likely at least in part explained by variable rates of concerted evolution among avian taxa. Whether supposedly genomic properties (such as interspecific recombination rate variation) or differing genomic structures of the MHC region among orders are involved in determining the rates of concerted evolution remains to be investigated. Gene conversion, the form of recombination driving concerted evolution, occurs predominantly between repeated sequences (including duplicate genes) situated in physically close genomic locations [31]. Variation in the proximity of MHCIIB paralogs could, therefore, cause rates of concerted evolution to vary among taxa. However, as in most vertebrates [34], avian MHCIIB genes are typically strongly linked (e.g. [35]). Apart from passerines [33], in the bird species for which the genomic structure of the MHC region is known, MHCIIB paralogs are typically situated at about the same distance of approximately five kilobases [24, 25, 36–38]. Other structural genomic features with possible effects on the rates of gene conversion could include the presence of MHC class IIA (MHCIIA) genes in- between MHCIIB paralogs. In crested ibis – the only bird species with both MHCIIB lineages for which the genomic structure of the MHC is known – MHCIIB and MHCIIA are tightly linked and duplicated as a unit in tandem [24, 25] such as in mammals, whereas in galliforms MHCIIA genes are situated outside the MHC region [39]. In conclusion, determining the extent to which avian MHCIIB lineages were masked by concerted evolution or lost by birth-death evolution, and the role of genomic MHC structure in determining rates of concerted evolution will require comparative MHC genomic studies that examine the physical position of MHCIIB genes within the MHC region in a range of species.

## Conclusions

We found that two ancient MHCIIB lineages evolved prior to the radiation of all extant birds >100 mya and that concerted evolution has contributed to the erosion of the phylogenetic signal of the duplication history to a varying degree in different bird orders.

The old age of the avian MHCIIB lineages may suggest that they have orthologs in as far relatives as mammals. Although the high evolutionary rates of MHCIIB genes hinder the identification of orthology across such vast timescales, MHCIIA genes may provide some insight on this question. In mammals, MHCIIA genes usually duplicated in tandem with MHCIIB genes [40] and their lower rates of evolution have previously enabled the establishment of orthology between chicken MHCII genes (*DAB2* lineage) and the mammalian *DR* lineage [39]. The isolation of avian MHCIIA sequences in species exhibiting both MHCIIB lineages might, therefore, provide an avenue to identify mammalian orthologs of the avian *DAB1* region. Together with the study of the genomic architecture of the MHC region in such species, this approach may provide insights into the evolution of vertebrate adaptive immunity over unprecedented timescales.

Our results provide a striking example of how concerted evolution may mask the evolutionary origins of gene lineages, and lead to patterns that potentially mimic gene loss and birth-death evolution. This raises questions regarding the extent to which similar processes may have masked the evolutionary history of multigene families in other taxonomic groups. Future analyses of the genomic structure of the MHC in bird species at different evolutionary stages along this process will provide deeper insights into the relative contributions of birth-death processes and concerted evolution in the long-term evolution of the avian MHCIIB.

## Methods

### Identification of avian MHCIIB lineages

To screen for the sequence signatures specific to the avian *DAB1* and *DAB2* MHCIIB lineages situated in MHCIIB exon 3 we first compiled an alignment of this region from as many avian species and orders as possible. To this end, we performed blast searches of an owl exon 3 sequence (GenBank accession no. EF641251) against all bird sequences in the GenBank DNA sequence database using the blastn algorithm. Sequence hits from other genes than MHCIIB and with a sequence identity inferior of 80% were removed. We also removed poorly/erroneously aligning sequences retrieved from genome assemblies that are based on short-read sequencing, as multigene families are prone to be collapsed during the assembly. GenBank accession numbers of the used sequences are provided in Additional file 10. The remaining sequences were aligned separately for each intron and exon using MAFFT 7 [41] with default settings on the MAFFT alignment server (http://mafft.cbrc.jp/alignment/server). Alignments are provided in Additional file 11.

Within this alignment, we then manually screened for the presence of the sequence signatures characteristic of the alternative MHCIIB lineages [9, 13] to determine the most recent common ancestor in birds that carried copies of both lineages. In addition, we performed phylogenetic reconstructions based on the first 220 bp of exon 3 (which were available for a large proportion of the species). The GTR + G nucleotide substitution model was evaluated as the best by jModeltest 2.1.7 [42] according to the Akaike information criterion [43, 44]. Bayesian phylogenetic reconstructions were then performed using MrBayes 3.2 [45]. Bayesian analyses were run with four chains for 5 × 10^6^ generations with sequences from tuatara (*Sphenodon punctatus*, accession number DQ124232) and Chinese alligator (*Alligator sinensis*, XM_006036594) as outgroups. Trees were sampled every 1000 generations. Posterior distributions were examined in Tracer 1.4 [46]. The first 25% of the topologies were discarded as burnin. Phylogenetic networks were computed in SplitsTree 4.13.1 [47] based on uncorrected p-distances. Moreover, we performed Bayesian phylogenetic reconstructions and estimated phylogenetic networks based on the 16 sites previously recovered to reflect MHCIIB duplication history [9, 14] using the same settings as outlined for exon 3.

### Screening for additional sites reflecting duplication history

To evaluate whether sites other than the 16 scattered across the 5′-end of MHCIIB exon 3 reflect duplication history of avian MHCIIB genes, we compiled a data set comprising MHCIIB sequences spanning MHCIIB exon 2 to exon 3 from 192 bird species from the GenBank database. These data included sequences that we previously isolated from 37 species from 13 orders with special attention to isolate sequences of both MHCIIB lineages where possible [28]. Sequences from tuatara were added as outgroups (see above). Sequences were aligned using MAFFT 7 [41] with E-INS-i settings recommended for sequences with long unalignable regions, such as expected for MHCIIB introns. To avoid difficulties with alignment due to repeat regions or transposable elements (TE), we ran CENSOR [48] to mask repeats and TEs in introns prior to alignment. The detected repeats and TEs were only found in single species and thus harboured no phylogenetic signal.

To identify sites that trace the duplication history of avian MHCIIB genes, we applied the hypothesis-free approach implemented in Saguaro [29], which applies a combination of a hidden Markov model (HMM) and a self-organising map (SOM) to characterize local phylogenetic relationships (‘cacti’) among aligned sequences. To determine the number of cacti that captures the phylogenetic histories contained in the alignment, we ran Saguaro with its default parameters for 2, 5, 10, and 15 iterations (each iteration a new cactus is proposed). The maximum number of sites reflecting duplication history was reached with five iterations. Results obtained with ≥5 iterations were all congruent and are not reported here.

We then determined which cacti might reflect duplication history. Within such cacti, sequences from the same species but from alternative lineages are expected to cluster separately and distant from each other, in each of the respective lineages. In contrast, for cacti that represent concerted evolution, a species’ sequences are expected to cluster close to each other. Therefore, the cross-species average of within-species’ pairwise distance among sequences should be higher for cacti reflecting duplication history than for cacti reflecting concerted evolution. Because the average pairwise distance among sequences for a given species would be biased by the number of sequences available for this species, and the number of sequences stemming from alternative paralogs, we retrieved the two most distant sequences of each species within a given cactus, and for each cactus estimated the mean of these across all species. Mean values were then normalized across cacti to compare values among runs with different numbers of iterations. For this, pairwise distances were extracted from cacti using the APE package [49] in R.

Finally, we concatenated the sites for which cacti reflecting duplication history were identified (Additional files 6A and 7) and performed phylogenetic reconstructions using MrBayes 3.2.0 [45] with a GTR + G nucleotide substitution model that was found to best fit the data using jModelTest2 [42] based on the Akaike information criterion [43], and estimated a phylogenetic network using SplitsTree 4.13.1 [47]. Running parameters were the same as for the phylogenetic analyses presented above.

## Additional files

Additional file 1: Intron-exon structure of avian MHCIIB genes. Main functions of the domains encoded by each exon are annotated. Approximate lengths of exons and introns in number of base pairs are indicated. Intron length is very variable and in many species not known; indicated is the range of intron lengths of MHCIIB sequences isolated in [28]. (PDF 312 kb)

Additional file 2: Phylogenetic relationships of MHCIIB exon 3 sequences. An easier to read version of this tree with clusters of sequences from the same order collapsed is provided in Fig. 2. Label colors depict similarity to the *DAB1* (blue) and *DAB2* (green) MHCIIB lineages. (PDF 728 kb)

Additional file 3: Neighbor-net network of MHCIIB exon 3 sequences. *DAB1* and *DAB2* clusters are highlighted in green and blue respectively. Orders contained in the main clusters are indicated. Orders with sequences distributed all over the cluster are indicated closer to the border. Orders with sequences in both clusters are highlighted with font the color of the other cluster. To read detailed labels, please zoom into the figure. (PDF 5114 kb)

Additional file 4: Phylogenetic relationships based on the 16 sites previously identified to reflect duplication history [9]. Bayesian posterior probabilities are provided for all nodes with support >50 and for the two main clusters deflecting *DAB1* and *DAB2*, respectively. Redundant sequences within orders were removed prior to phylogenetic reconstruction. The consensus tree taking into account all compatible branches is shown. (PDF 251 kb)

Additional file 5: Neighbor-net network based on the 16 sites originally reported from owls to reflect duplication history. *DAB1* and *DAB2* clusters are highlighted in green and blue respectively. Orders contained in the main clusters are indicated. Orders with sequences distributed all over the cluster are indicated closer to the border. Orders with sequences in both clusters are highlighted with font the color of the other cluster. To read detailed labels, please zoom into the figure. (PDF 3684 kb)

Additional file 6: Cacti resulting from Saguaro analyses [29] with five iterations. A, cacti with large distances among species’ most distant MHCIIB sequences (Additional file 7), representing duplication history (cacti 3 and 5). B, cacti with small distances among species’ most distant MHCIIB sequences. (PDF 7426 kb)

Additional file 7: Table: Mean pairwise distances between species’ most distant sequences for each cactus. High values, such for cactus 3 and 5, indicate cacti reflecting duplication history. (DOCX 25 kb)

Additional file 8: Phylogenetic relationships based on the ten sites identified to reflect duplication history using Saguaro [29]. Bayesian posterior probabilities are provided for all nodes with support >50 and for the two main clusters deflecting *DAB1* and *DAB2*, respectively. Redundant sequences within orders were removed prior to phylogenetic reconstruction. The consensus tree taking into account all compatible branches is shown. (PDF 1058 kb)

Additional file 9: Neighbor-net network at the ten sites identified to reflect duplication history using Saguaro [29]. *DAB1* and *DAB2* clusters are highlighted in green and blue respectively. Orders contained in the main clusters are indicated. Orders with sequences distributed all over the cluster are indicated closer to the border. Orders with sequences in both clusters are highlighted with font the color of the other cluster. To read detailed labels, please zoom into the figure. (PDF 4157 kb)

Additional file 10: GenBank accession numbers of MHCIIB sequences retained for analysis. (XLSX 23 kb)

Additional file 11: Fasta format alignments of all MHCIIB sequences used for analysis. Separate alignments are provided for each exon and intron comprised between exon 1 to exon 4. Two alignments are provided for each region, one including all sequences retrievable and one including all accessions for which exon 3 was available. For the latter, all accession were retained for all regions, even if no sequence data was available. (ZIP 354 kb)
