# Supplementary figures and images for: 100 million years of multigene family evolution: origin and evolution of the avian MHC class IIB
Source: BMC Genomics. 2017 Jun 13;18:460. doi: 10.1186/s12864-017-3839-7 (PMC5470263; doi:10.1186/s12864-017-3839-7)

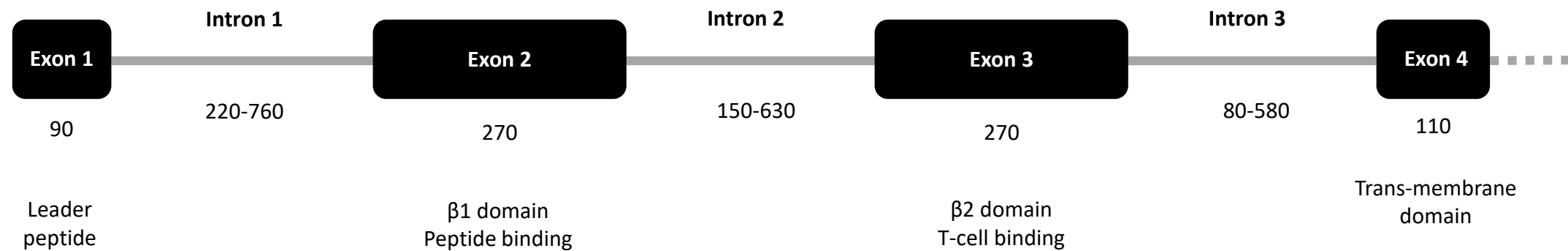

Supplement: Supplementary file 1 — Intron-exon structure of avian MHCIIB genes. Main functions of the domains encoded by each exon are annotated. Approximate lengths of exons and introns in number of base pairs are indicated. Intron length is very variable and in many species not known; indicated is the range of intron lengths of MHCIIB sequences isolated in [28]. (PDF 312 kb) [file 12864_2017_3839_MOESM1_ESM.pdf]

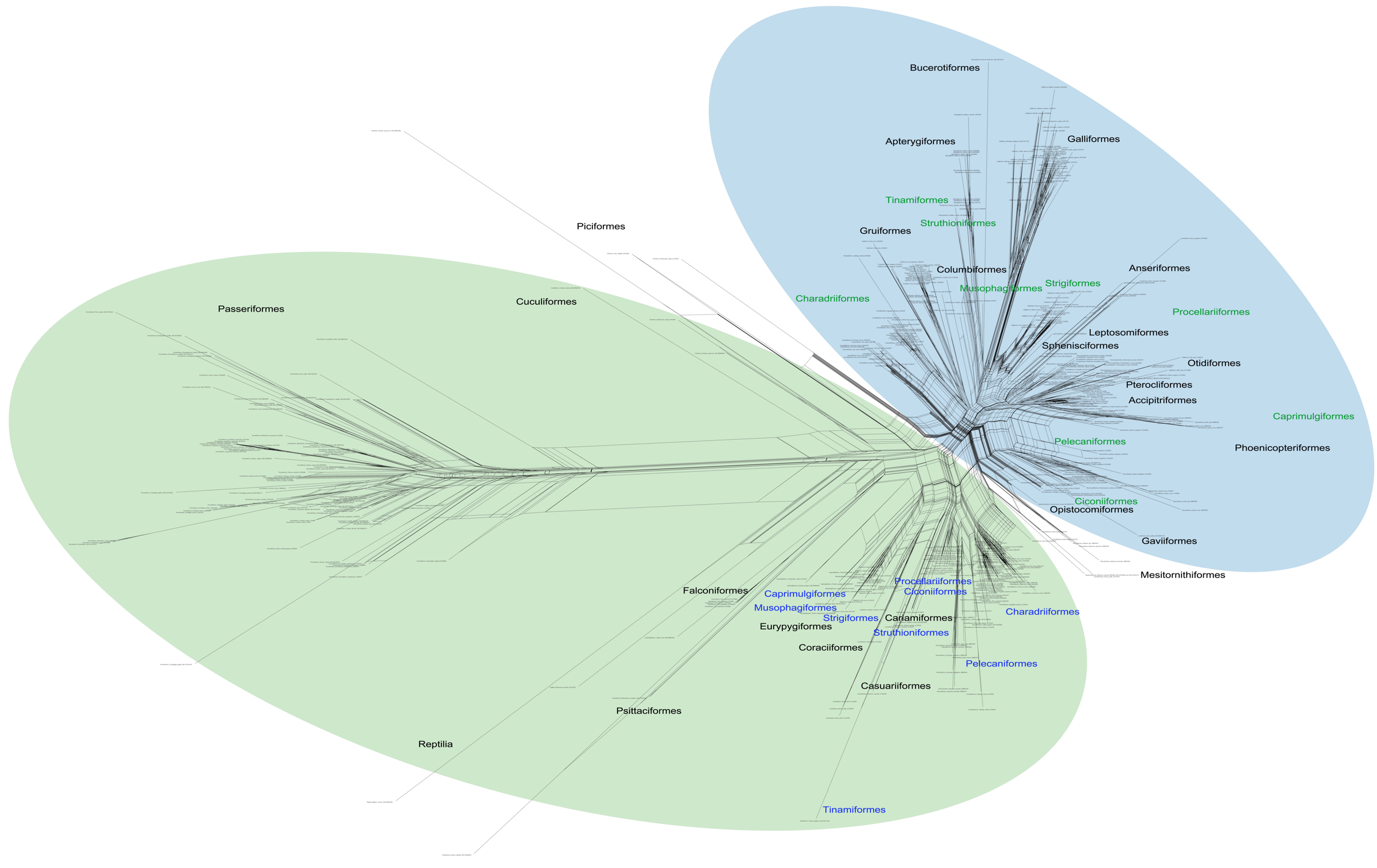

Supplement: Supplementary file 3 — Neighbor-net network of MHCIIB exon 3 sequences. DAB1 and DAB2 clusters are highlighted in green and blue respectively. Orders contained in the main clusters are indicated. Orders with sequences distributed all over the cluster are indicated closer to the border. Orders with sequences in both clusters are highlighted with font the color of the other cluster. To read detailed labels, please zoom into the figure. (PDF 5114 kb) [file 12864_2017_3839_MOESM3_ESM.pdf]

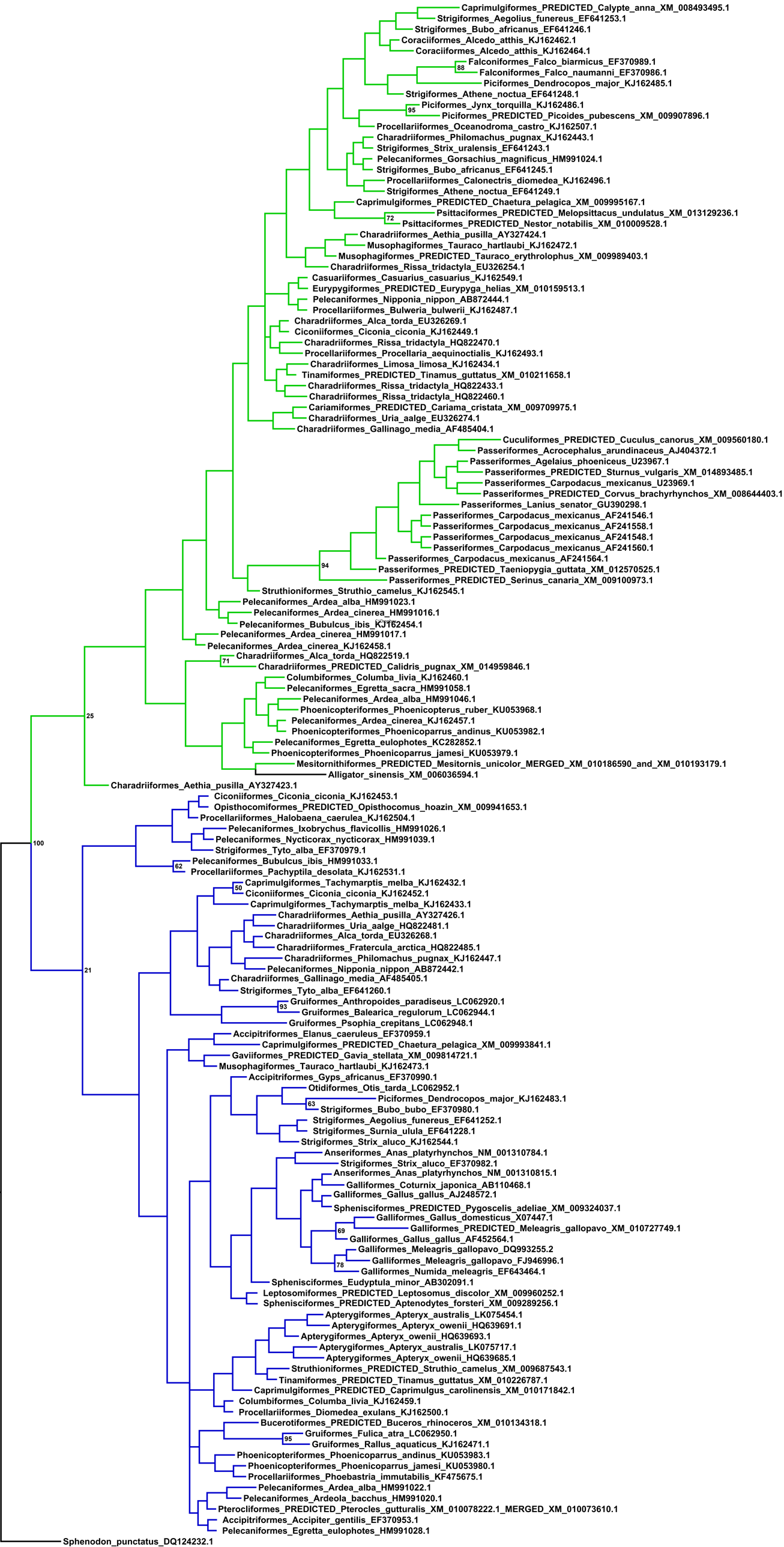

Supplement: Supplementary file 4 — Phylogenetic relationships based on the 16 sites previously identified to reflect duplication history [9]. Bayesian posterior probabilities are provided for all nodes with support >50 and for the two main clusters deflecting DAB1 and DAB2, respectively. Redundant sequences within orders were removed prior to phylogenetic reconstruction. The consensus tree taking into account all compatible branches is shown. (PDF 251 kb) [file 12864_2017_3839_MOESM4_ESM.pdf]

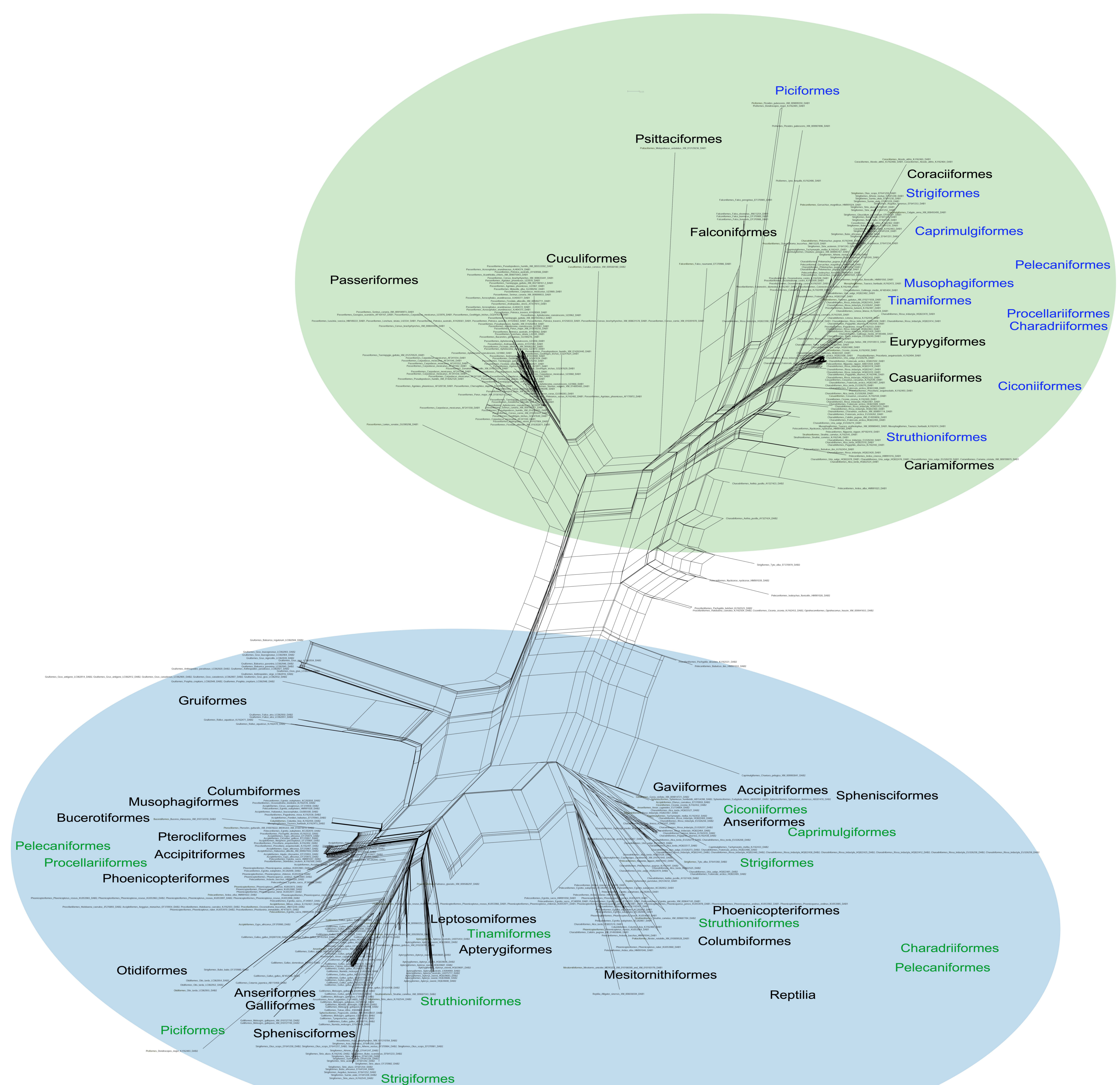

Supplement: Supplementary file 5 — Neighbor-net network based on the 16 sites originally reported from owls to reflect duplication history. DAB1 and DAB2 clusters are highlighted in green and blue respectively. Orders contained in the main clusters are indicated. Orders with sequences distributed all over the cluster are indicated closer to the border. Orders with sequences in both clusters are highlighted with font the color of the other cluster. To read detailed labels, please zoom into the figure. (PDF 3684 kb) [file 12864_2017_3839_MOESM5_ESM.pdf]

A

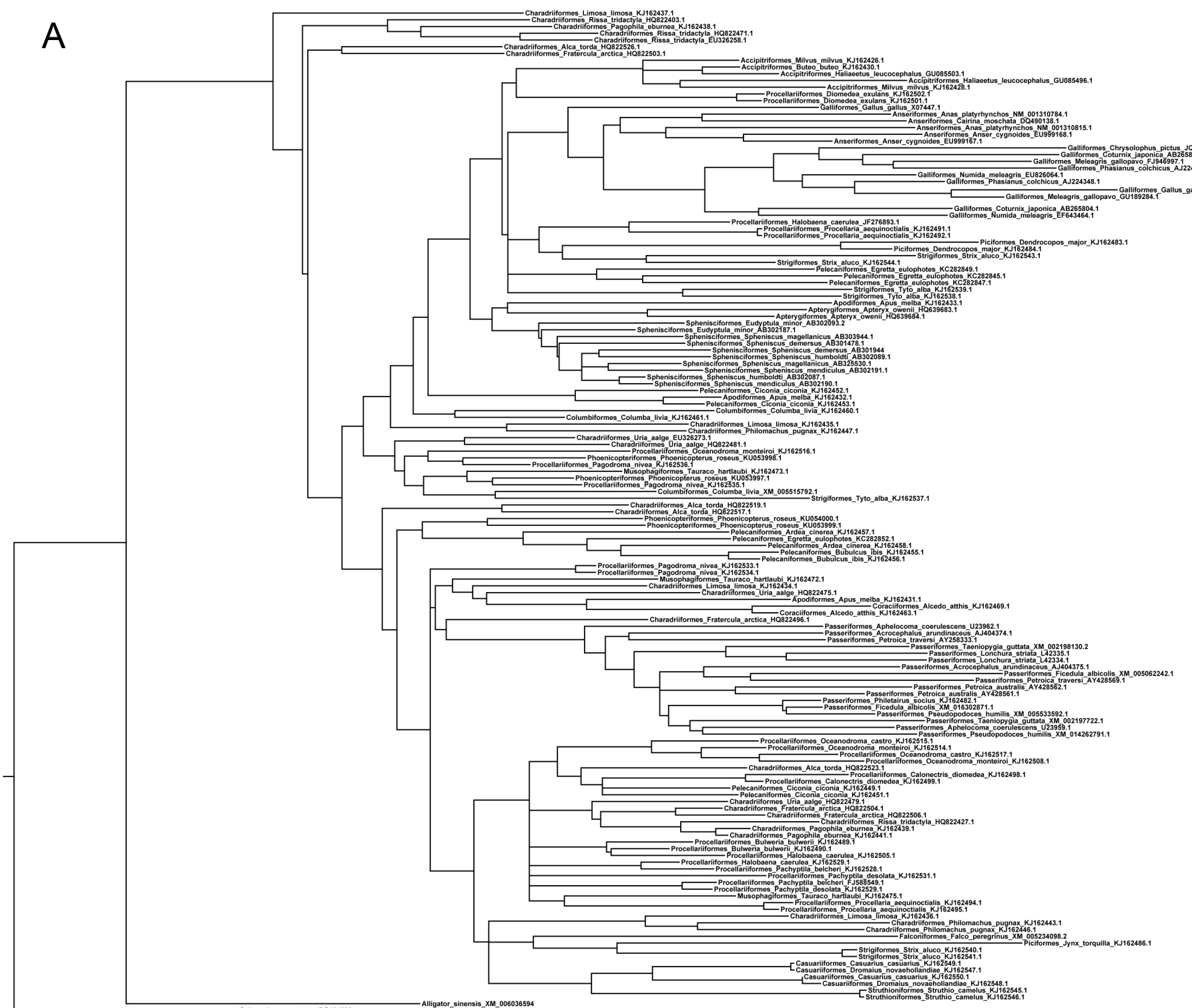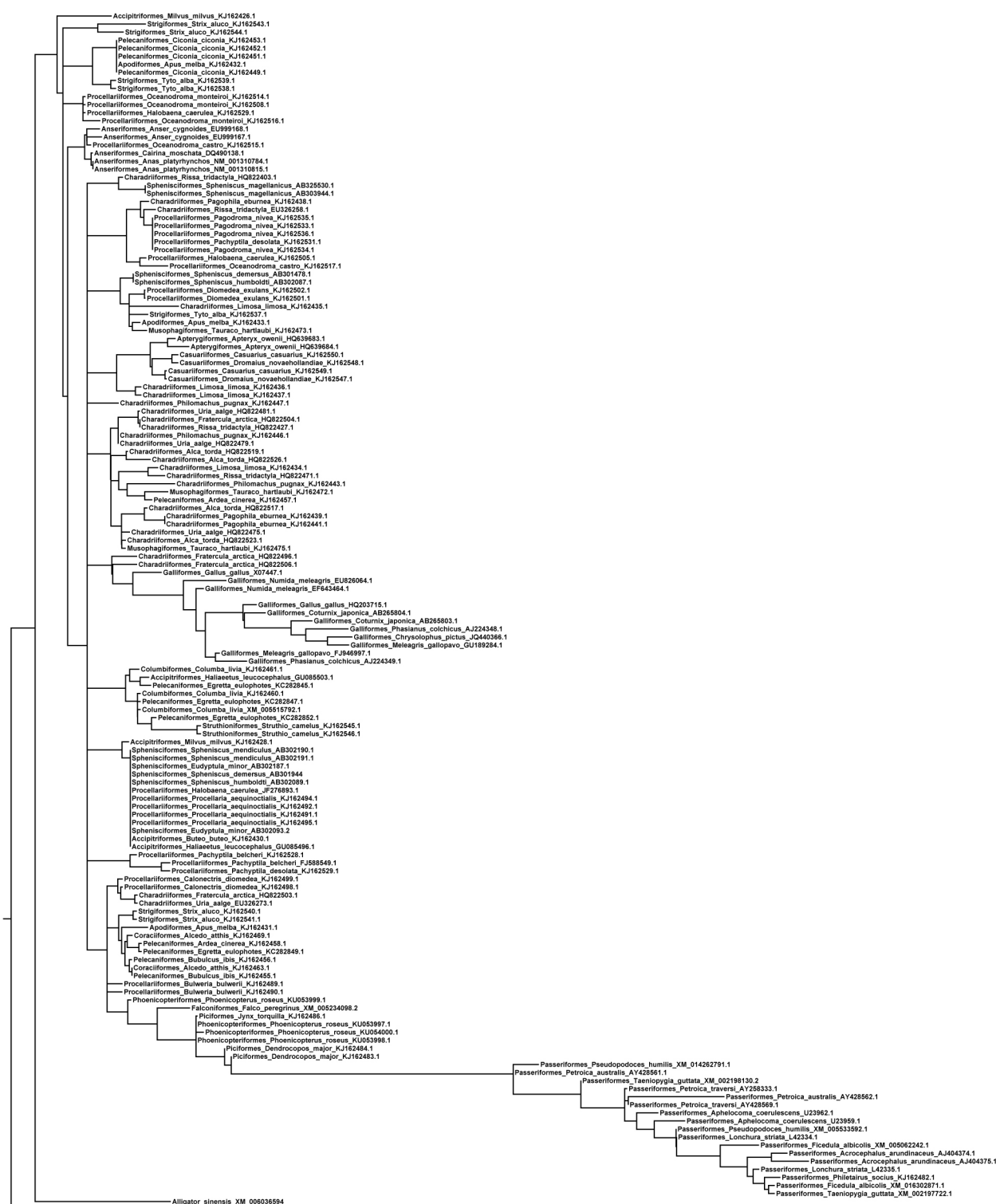

# B

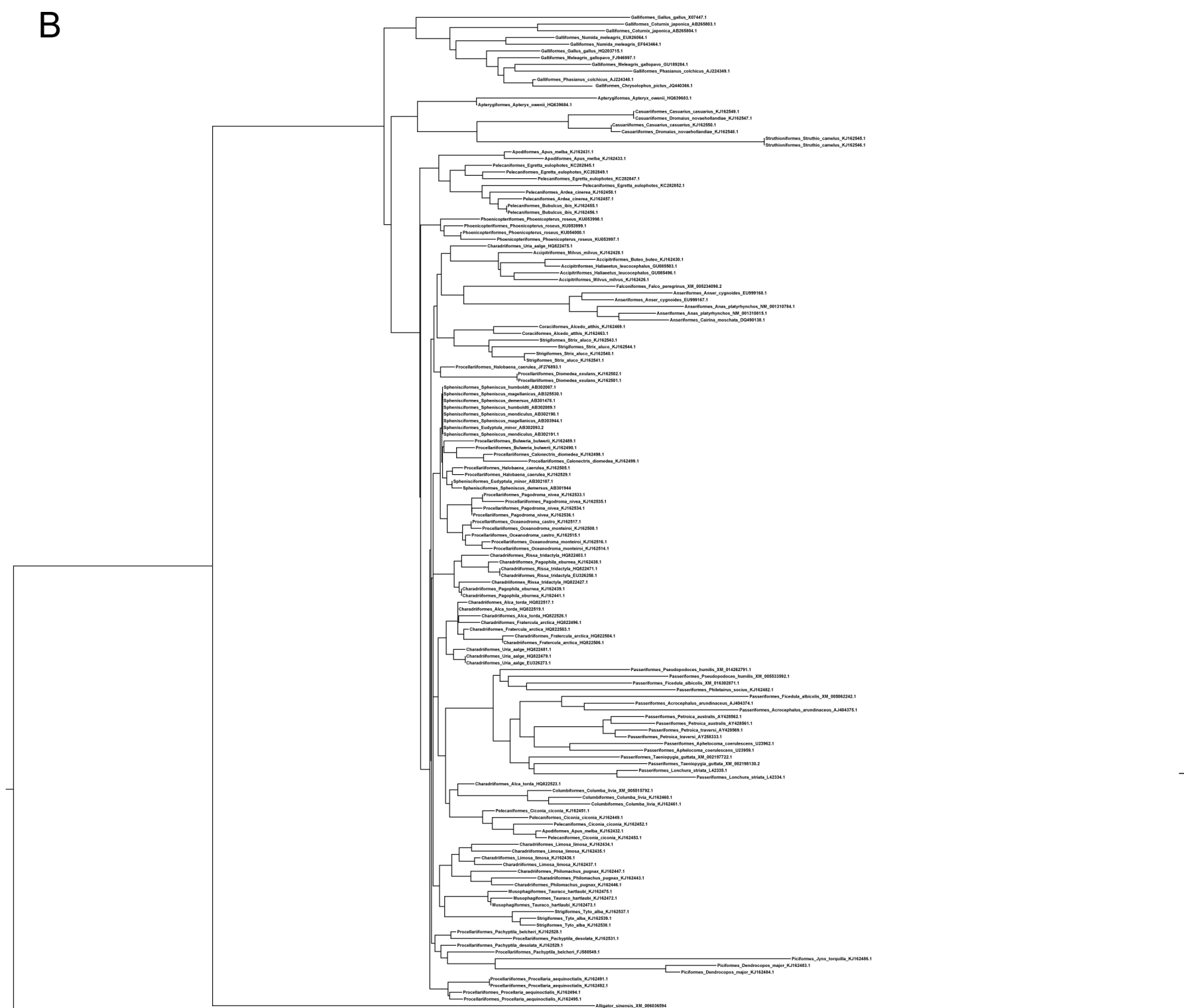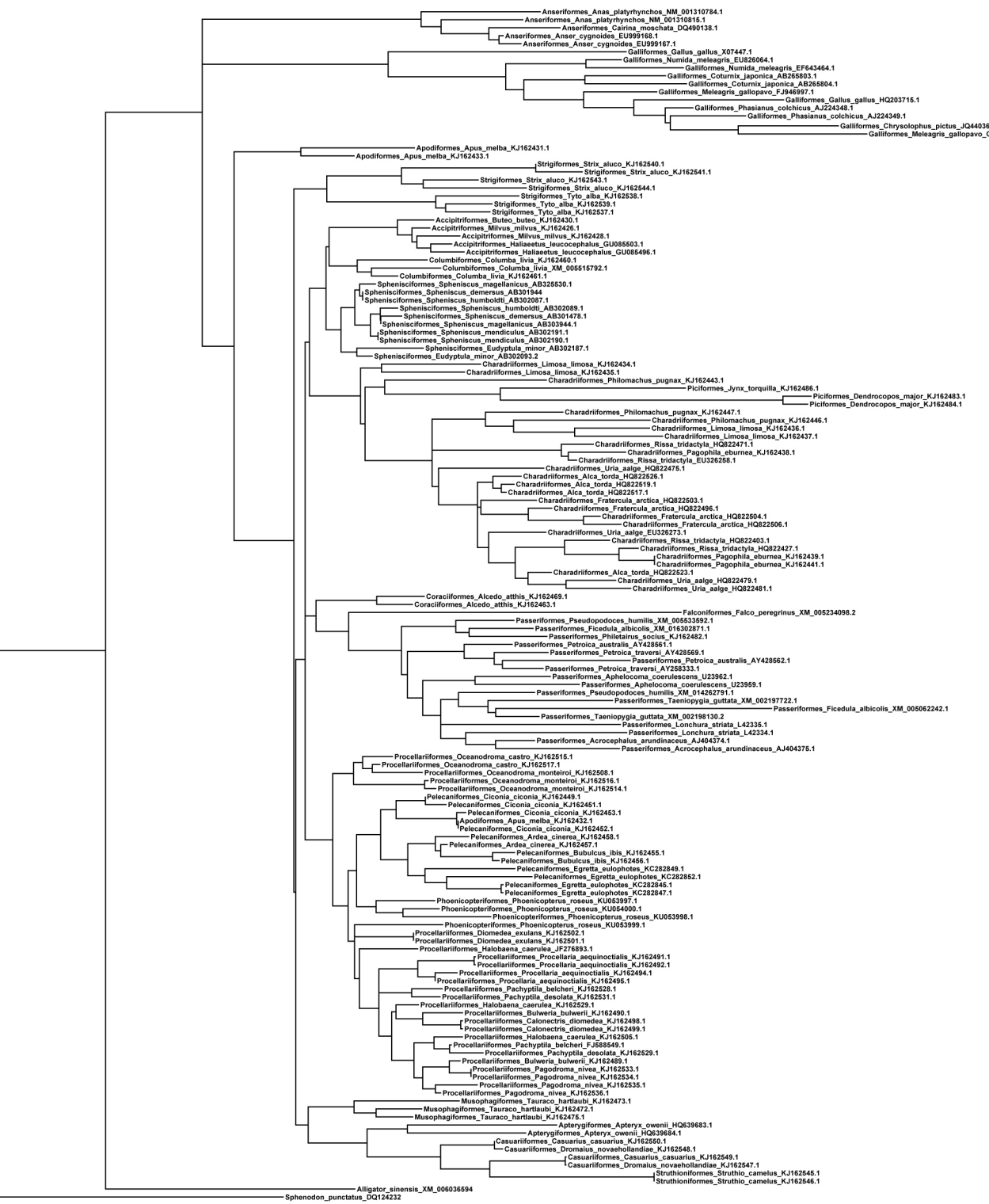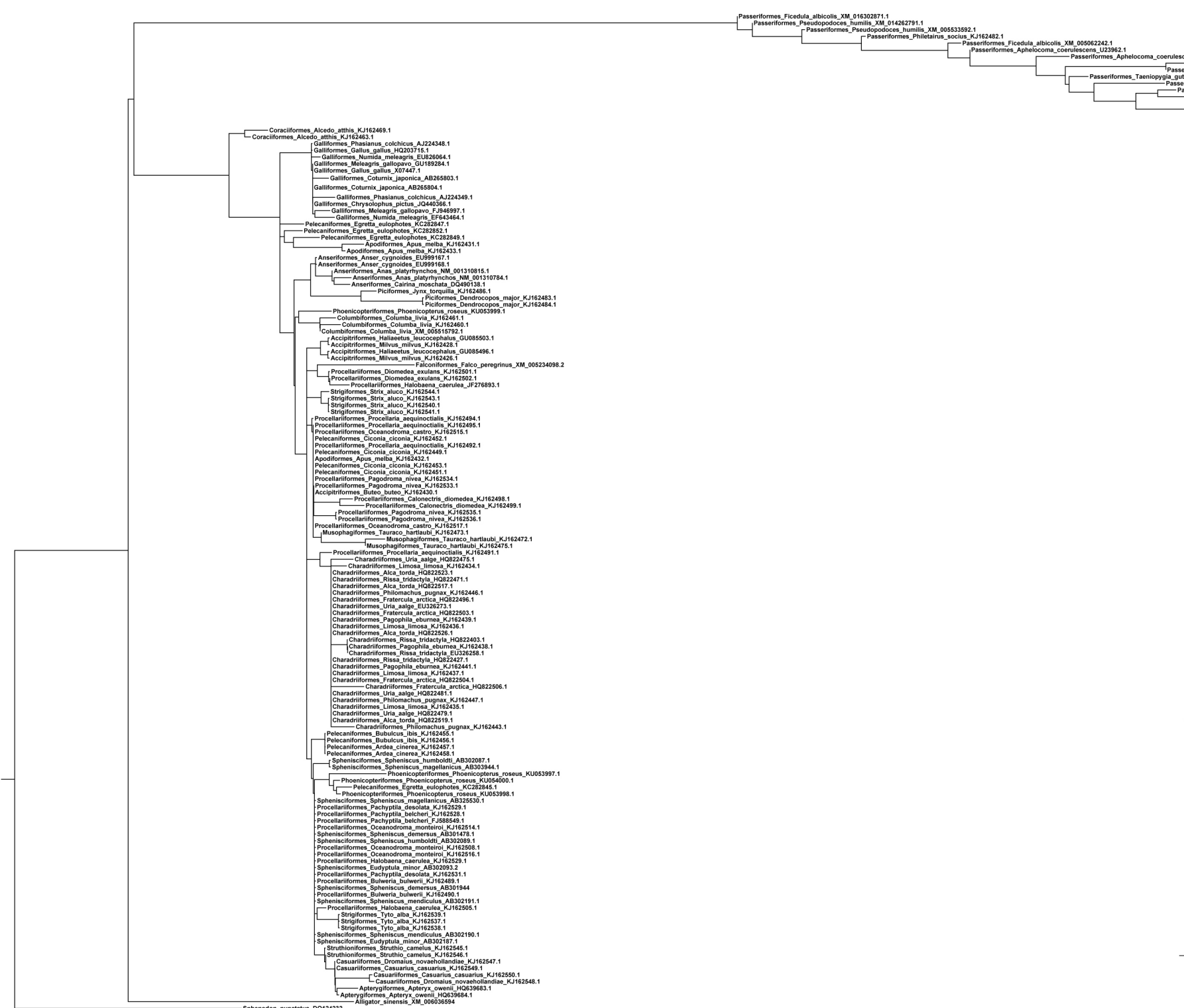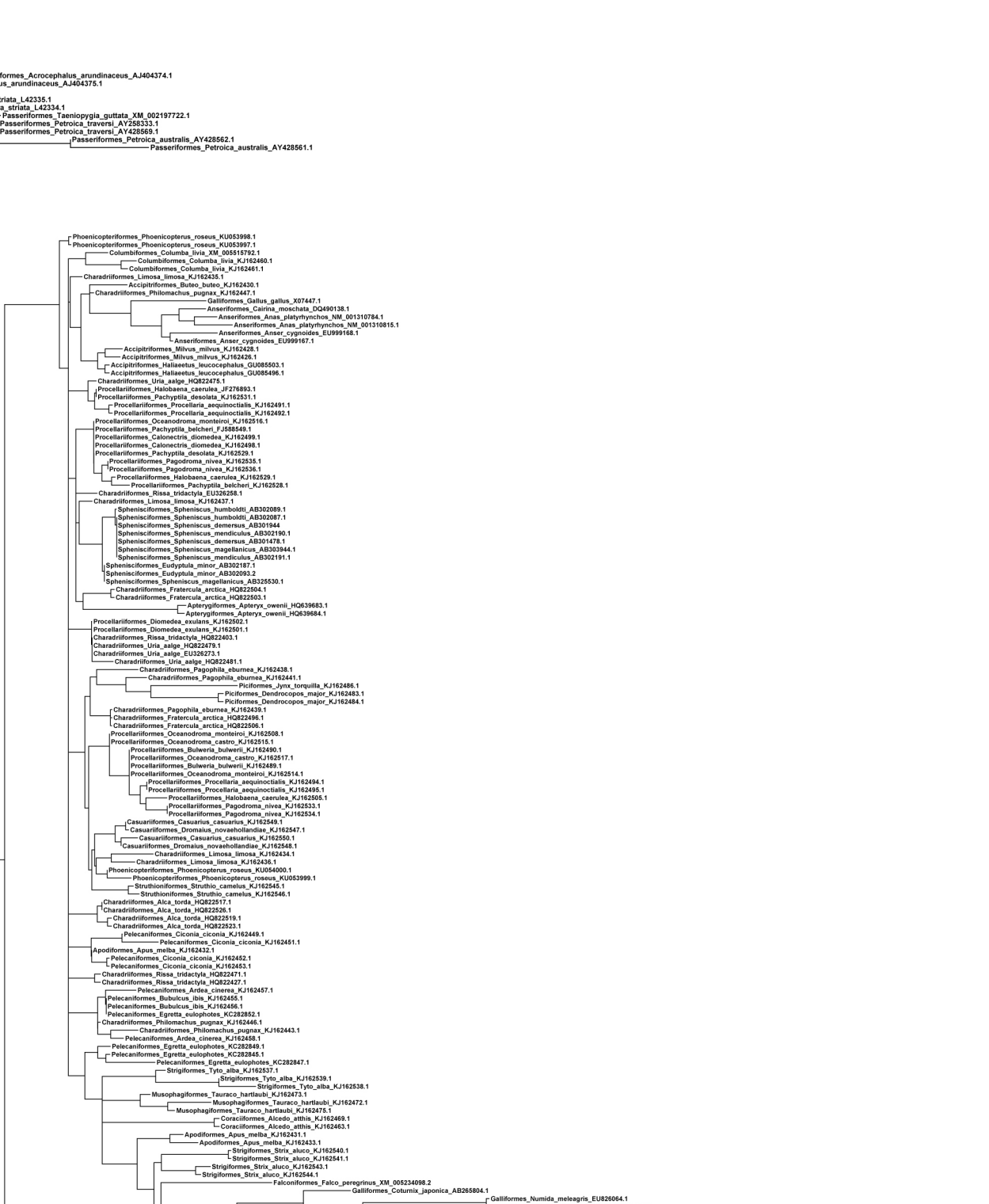

Supplement: Supplementary file 6 — Cacti resulting from Saguaro analyses [29] with five iterations. A, cacti with large distances among species’ most distant MHCIIB sequences (Additional file 7), representing duplication history (cacti 3 and 5). B, cacti with small distances among species’ most distant MHCIIB sequences. (PDF 7426 kb) [file 12864_2017_3839_MOESM6_ESM.pdf]

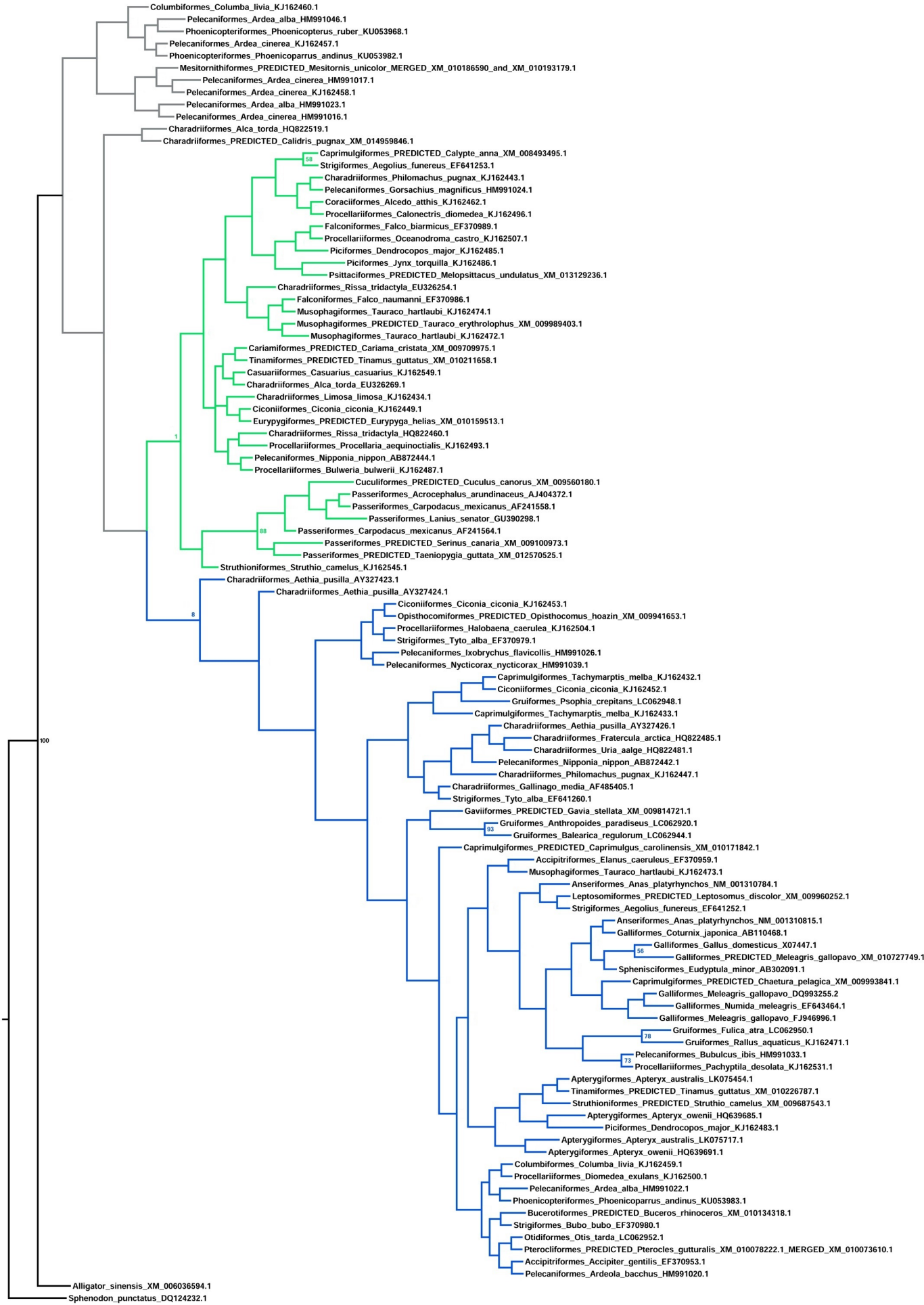

Supplement: Supplementary file 8 — Phylogenetic relationships based on the ten sites identified to reflect duplication history using Saguaro [29]. Bayesian posterior probabilities are provided for all nodes with support >50 and for the two main clusters deflecting DAB1 and DAB2, respectively. Redundant sequences within orders were removed prior to phylogenetic reconstruction. The consensus tree taking into account all compatible branches is shown. (PDF 1058 kb) [file 12864_2017_3839_MOESM8_ESM.pdf]

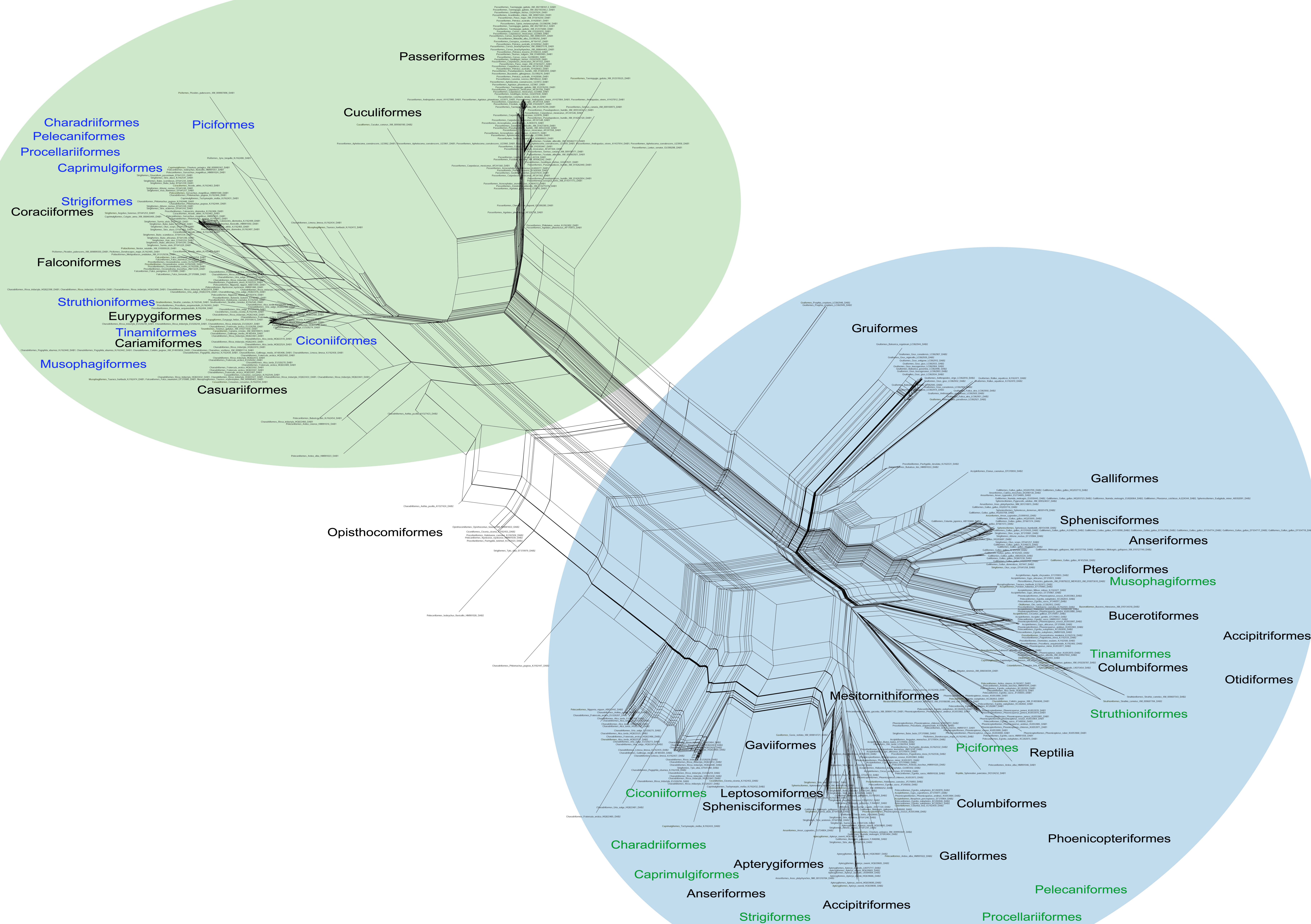

Supplement: Supplementary file 9 — Neighbor-net network at the ten sites identified to reflect duplication history using Saguaro [29]. DAB1 and DAB2 clusters are highlighted in green and blue respectively. Orders contained in the main clusters are indicated. Orders with sequences distributed all over the cluster are indicated closer to the border. Orders with sequences in both clusters are highlighted with font the color of the other cluster. To read detailed labels, please zoom into the figure. (PDF 4157 kb) [file 12864_2017_3839_MOESM9_ESM.pdf]
