# Supplementary material for: 100 million years of multigene family evolution: origin and evolution of the avian MHC class IIB
Source: BMC Genomics. 2017 Jun 13;18:460. doi: 10.1186/s12864-017-3839-7 (PMC5470263; doi:10.1186/s12864-017-3839-7)
Supplement: Supplementary file 7 — Table: Mean pairwise distances between species’ most distant sequences for each cactus. High values, such for cactus 3 and 5, indicate cacti reflecting duplication history. (DOCX 25 kb) [file 12864_2017_3839_MOESM7_ESM.docx]

| **Cactus** | **Mean** | **Normalized Mean** |
| --- | --- | --- |
| 0 | 0.06 | -0.75 |
| 1 | 0.13 | -0.24 |
| 2 | 0.04 | -0.89 |
| 3 | 0.43 | 1.77 |
| 4 | 0.11 | -0.41 |
| 5 | 0.25 | 0.53 |
