# Supplementary material for: 100 million years of multigene family evolution: origin and evolution of the avian MHC class IIB
Source: BMC Genomics. 2017 Jun 13;18:460. doi: 10.1186/s12864-017-3839-7 (PMC5470263; doi:10.1186/s12864-017-3839-7)

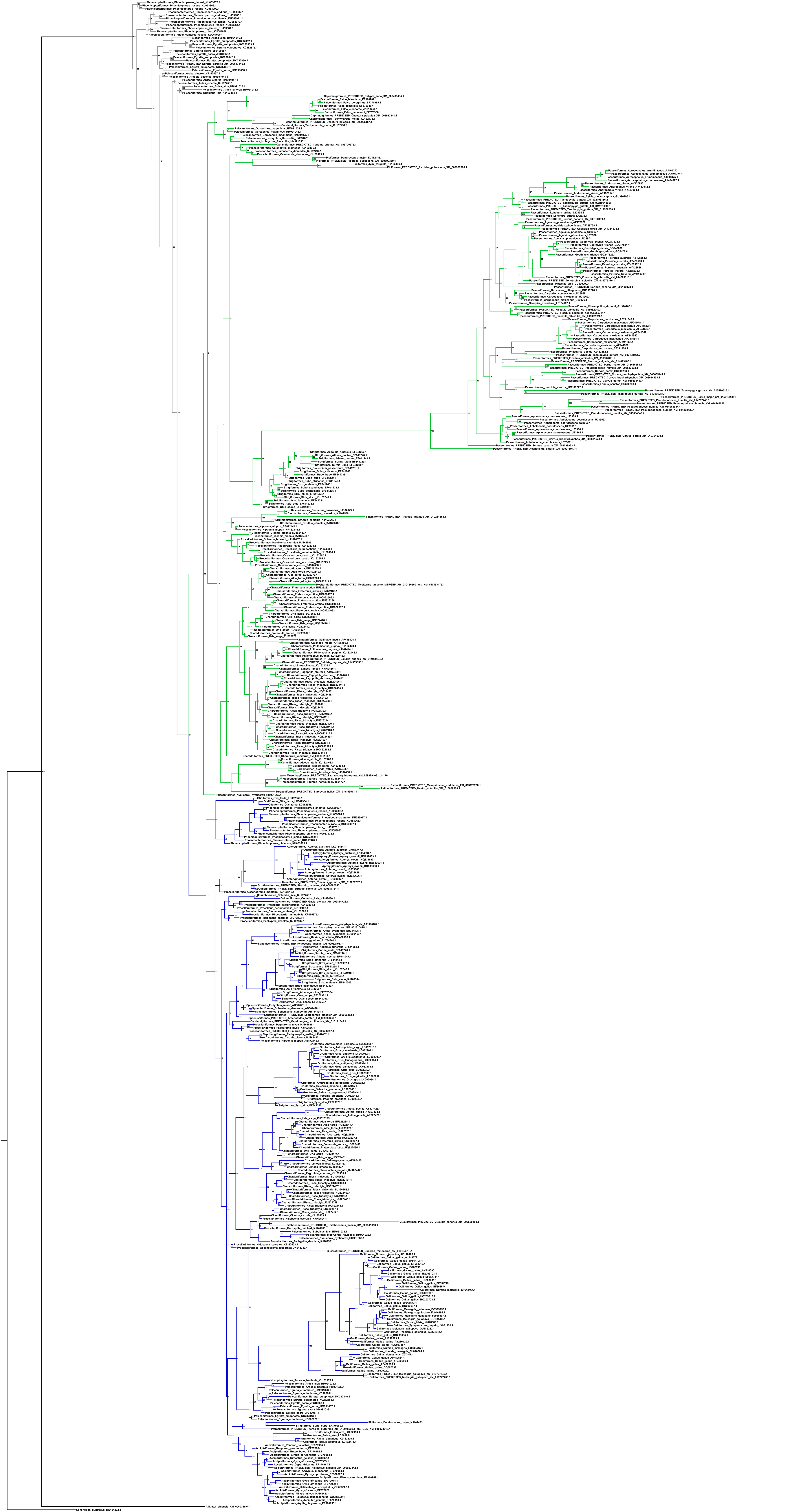

Supplement: Supplementary file 2 — Phylogenetic relationships of MHCIIB exon 3 sequences. An easier to read version of this tree with clusters of sequences from the same order collapsed is provided in Fig. 2. Label colors depict similarity to the DAB1 (blue) and DAB2 (green) MHCIIB lineages. (PDF 728 kb) [file 12864_2017_3839_MOESM2_ESM.pdf]
